# Intraoperative and Long-Term Impedance Changes and Electrode Malfunctions in Cochlear Implant Recipients

**DOI:** 10.3390/jcm15103662

**Published:** 2026-05-10

**Authors:** Muhammed Dagkiran, Ilda Tanrisever Pehlivan

**Affiliations:** Faculty of Medicine, Department of Otolaryngology and Head and Neck Surgery, Campus of Balcali, Cukurova University, 01330 Saricam, Adana, Turkey; muhammeddagkiran@gmail.com

**Keywords:** cochlear implant, impedance telemetry, electrode malfunction, open circuit, short circuit

## Abstract

**Objective:** This study aimed to investigate intraoperative and long-term impedance changes in cochlear implant recipients and to evaluate the prevalence and course of electrode circuit malfunctions. **Methods:** A retrospective longitudinal observational study was conducted on 358 ears that underwent cochlear implantation between October 2023 and June 2024. Intraoperative and postoperative impedance telemetry measurements were obtained at 1, 2, 6, and 12 months. Electrode arrays were analyzed according to their positions (basal, medial, apical), and electrode malfunctions were classified as open circuit (OC) or short circuit (SC). **Results:** Impedance values significantly increased from intraoperative to first activation measurements, reached their peak at the 1st postoperative month, and gradually decreased, stabilizing after the 6th month (*p* < 0.05). Electrode malfunctions were detected in 26 of 358 devices (7.2%), with 22 OCs (84.7%) and 4 SCs (15.3%). While 4 of the 22 OC cases improved within the first month after surgery, no improvement was observed in SC cases during the 12-month follow-up period. **Conclusions:** Impedance telemetry is useful for assessing electrode integrity intraoperatively and for monitoring changes over time during follow-up. These findings highlight the dynamic nature of impedance changes, particularly during the first 6 months, and underscore the value of regular follow-up measurements and individualized programming.

## 1. Introduction

Cochlear implants (CIs) are devices that convert mechanical acoustic energy into electrical signals, directly stimulating the cochlear nerve. Medical, psychological, audiological, language development, and radiological evaluations are required for patient selection. Thus, efforts are made to improve the quality of life for individuals with hearing loss [1,2].

As the eligibility criteria for CI recipients expand, the number of patients undergoing implantation and the incidence of individual electrode circuit failures will predictably increase. Despite significant advances between electrode generations, the sheer number of patients implanted each year means that individual electrode circuit failures are inevitable [3,4,5].

Intraoperative electrophysiological measurements are commonly performed to detect potential complications and provide feedback to the surgeon and audiologist. These tests provide comprehensive information about the integrity and electrical status of the electrode. In the postoperative period, establishing electrical thresholds to reveal acoustic reflexes and central nervous system responses provides important information for fitting the speech processor. These measurements should be performed using telemetry-based methods that enable communication with the implant system, such as impedance telemetry, neural response telemetry (NRT), the electrically stimulated stapes reflex test (ESRT), and the trans-impedance matrix test (TIM).

The most commonly used parameter during implantation and speech processor programming is impedance telemetry. Impedance refers to the resistance exhibited against the flow of energy in any medium. Electrode impedance is defined as the resistance to current flow between a reference electrode and the ground [6,7]. The impedance of an electrode reflects the electrical state of the electrode and the surrounding tissue environment. Intracochlear contents, bone, and soft tissue affect the overall impedance to varying degrees [8]. High impedance levels mean that more energy will be required to stimulate the system [9]. These impedance measurements not only verify the functionality of the implanted electrodes; they also provide guidance in adjusting the device’s clinical stimulation levels.

Although the general temporal pattern of impedance changes following cochlear implantation has been well established, important gaps remain in the detailed characterization of these changes in large, homogeneous cohorts and in the understanding of electrode malfunction behavior. In particular, there is limited high-resolution longitudinal data for widely used electrode arrays, as well as a lack of systematic comparison between different types of electrode circuit failures.

Therefore, the present study aims not only to describe impedance changes over time in a large cohort of cochlear implant recipients using a predominantly uniform electrode type (CI422), but also to provide a detailed analysis of the prevalence, anatomical distribution, and longitudinal course of electrode malfunctions. Special emphasis is placed on comparing the natural history of open-circuit (OC) and short-circuit (SC) failures, thereby offering clinically relevant insights into their expected evolution and management.

## 2. Materials and Methods

Our study was a retrospective longitudinal observational study conducted between October 2023 and June 2024 at the Department of Otolaryngology, Faculty of Medicine, Cukurova University. The study was approved by the Ethics Committee of Cukurova University at its meeting on 13 October 2023, with decision number 137. Ethics committee approval was obtained for all cases included in the study, and an “Informed Consent Form” was signed. This design reflects the repeated assessment of impedance measurements within the same individuals over multiple postoperative time points.

Our study included 333 patients aged 1–75 years with sensorineural hearing loss at an advanced or very advanced level, involving 358 ears. Patients were evaluated at the Audiology Unit of the Department of Otorhinolaryngology, Cukurova University Faculty of Medicine and were selected among those who had undergone cochlear implant surgery at the same clinic. Of the 358 devices included in the study, 15 had Nucleus CA (Contour Advance) electrodes, 337 had Nucleus CI422 (Slim Straight) electrodes, and 6 had Nucleus CI512 electrodes.

### 2.1. Inclusion Criteria for the Study

- The presence of advanced or profound sensorineural hearing loss;
- The pbsence of cochlear anomalies;
- The patient has not undergone revision surgery in the ear to be measured;
- No atypical findings are observed in the electrodes within the cochlea on the Stenvers Graph taken on the 1st postoperative day;
- The implant has not been removed for any reason, such as infection or trauma, during the postoperative period.

### 2.2. Equipment and Application

In our study, the instruments in the standard test battery were used during both the intraoperative and postoperative processes and did not require any additional equipment. This equipment consisted of one laptop computer with the Custom Sound (version 6.0) program, one pod used to connect the laptop to the cochlear sound processor, and one cochlear sound processor (Nucleus 6/CP 910) used for standard measurements and adjustments.

### 2.3. Data Collection Method

Intraoperative and postoperative impedance measurement: Impedances were measured at all electrodes after electrode placement during surgery and before measuring the electrically evoked compound action potential. Impedances were measured using the manufacturers’ default modes: common ground (CG) for cochlear devices for all 22 electrodes and all three monopolar modes (MP1, MP2, or MP1 + 2). Neural response telemetry and impedance telemetry were routinely performed intraoperatively for all patients. Impedance measurements were recorded in ohms, and electrodes with short circuits or open circuits were noted. The first stimulations were performed at 4 weeks postoperatively. The 1-month follow-up measurement corresponds to the first activation session performed approximately 4 weeks after surgery; therefore, these two terms refer to the same clinical time point. Follow-up appointments are typically scheduled at 1, 2, 6, and 12 months. Subsequent electrode impedance tests vary depending on the individual patient’s performance, but the majority are performed at least once a year. Only impedance telemetry measurements were included in the longitudinal analysis; other telemetry measures such as neural response telemetry (NRT) or electrically evoked stapedial reflex thresholds (ESRTs) were not evaluated serially in this study.

Impedance telemetry data from individuals who underwent cochlear implantation surgery were analyzed during the intraoperative and postoperative periods (1st, 2nd, 6th, and 12th months). To minimize variability in the study, a single brand of electrode (Cochlear, Nucleus model) was preferred. The electrode arrays were grouped based on the average impedance values of the electrodes: apical (16–22) electrodes, medial (8–15) electrodes, and basal (1–7) electrodes. Electrode circuit malfunctions with abnormal impedance detected were recorded as SC = short circuit and OC = open circuit. Electrode circuit abnormalities were identified using the manufacturer-defined impedance classification provided by CustomSound software version 6.0 (Cochlear Ltd., Sydney, NSW, Australia). Open circuits (OCs) and short circuits (SCs) were automatically flagged by the system based on internal impedance criteria. No additional or custom cutoff values were applied by the investigators. All classifications used for analysis were derived directly from the impedance telemetry reports generated by the software. According to the manufacturer, SC is identified when impedance falls below the predefined low-impedance threshold, and OC is identified when impedance exceeds the high-impedance threshold [10]. Malfunction changes were recorded in the postoperative period in individuals with observed electrode malfunctions. Furthermore, the prevalence of individual electrode malfunctions within the evaluated patient population was determined.

Improvement of an open circuit (OC) was defined as either complete or partial. Complete improvement was defined as normalization of impedance values into the manufacturer-defined normal range and removal of the OC flag by the impedance telemetry software. Partial improvement was defined as a decrease in impedance measurements toward the normal range without full normalization, such that the electrode was still classified as an OC.

## 3. Results

Our study was retrospective, and a total of 358 cochlear implant devices were evaluated in 333 patients. Of the total 333 patients included in the study, 25 (7.5%) underwent bilateral and 308 (92.5%) underwent unilateral cochlear implantation. Of the 308 patients with unilateral implants, 173 (51.9%) had the implant placed in the right ear and 135 (40.5%) in the left ear.

The mean chronological age of the patients at the time of data analysis was 15.9 years (range: 0.9–75.9 years), whereas the mean age at implantation was 6.4 years (range: 1.1–17.2 years), indicating a predominantly pediatric implantation cohort. Age-related factors may also influence impedance dynamics and electrode behavior; however, this aspect was not specifically analyzed in the present study.

All 358 devices evaluated in our study were Cochlear^TM^ brand (Cochlear Ltd., Macquarie, NSW, Australia) implants. Of these, 337 electrodes (94%) were Nucleus CI422 (Slim Straight), 15 (5%) were Nucleus CI24RE (Contour Advance), and 6 (1%) were Nucleus CI512 type.

### Changes in Impedance Values During the Intraoperative and Postoperative Periods

According to the Shapiro–Wilk test, impedance values were normally distributed at all time points (*p* > 0.05), and repeated measures ANOVA with within-subject factor (time) was therefore applied.

Overall impedance changes: Repeated measures ANOVA demonstrated a significant effect of time on impedance values across all electrodes (F = 25.27, *p* < 0.001), indicating that impedance increased from the intraoperative measurement to the first postoperative month, then gradually decreased and stabilized thereafter.

Basal electrodes: Basal electrode impedance values showed a significant change over time (F = 3.25, *p* = 0.007), with a peak at the first postoperative month followed by gradual stabilization.

Medial electrodes: Medial electrode impedance values also demonstrated a statistically significant change over time (F = 5.13, *p* < 0.001), although the magnitude of change was smaller compared to basal electrodes.

Apical electrodes: In contrast, apical electrode impedance values did not show a statistically significant change over time (F = 0.93, *p* = 0.459), despite a visible increase at the first postoperative month followed by a decline, suggesting relative stability in this cochlear region throughout follow-up.

**Figure 1:**
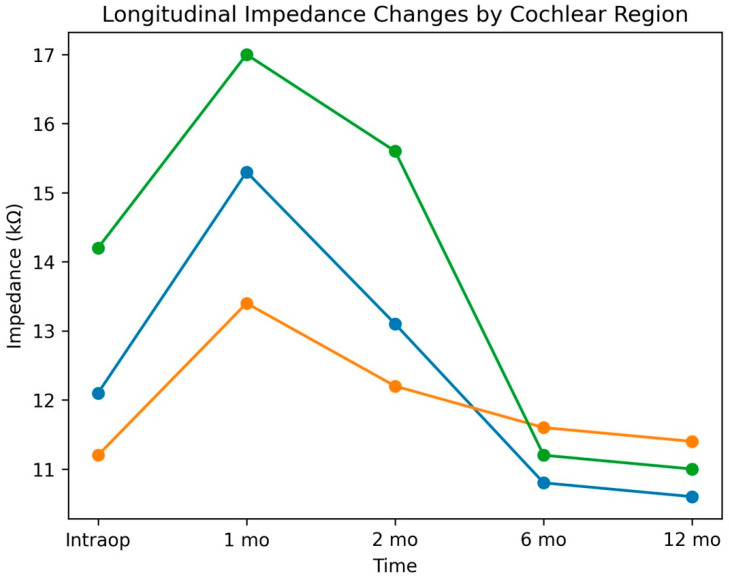
Longitudinal impedance changes in basal (blue), medial (orange), and apical (green) electrode groups.

The intraoperative, 1st, 2nd, 6th, and 12th month impedance values and significance levels for all electrodes included in the study, based on their positions in the cochlea, are listed in Table 1. These regional differences in longitudinal impedance trends are illustrated in Figure 1. According to the findings, impedance values reached their maximum value at the first activation (1st month) from the intraoperative process onwards, began to decline from this period, and remained stable from the 6th month onwards.

Of the 358 devices examined from the intraoperative process onwards, at least one electrode malfunction occurred in 26 (7.2%). Of the 26 devices with individual electrode malfunctions, 22 (84.7%) were found to have an open circuit (OC) and 4 (15.3%) had a short circuit (SC). Open-circuit electrode failures were evaluated using evoked compound action potential and the evoked stapes muscle reflex. In the patient with a multiple-short-circuit malfunction, the electrode was removed and reinserted, and then its position within the cochlea was checked using scopy. All malfunction data were recorded during the intraoperative phase. Of the devices with abnormal electrode malfunctions, 25 had Nucleus CI422 electrodes and 1 had a Nucleus CI24RE electrode.

When we grouped the abnormal electrodes according to their positions in the cochlea, we found that 14 (53%) were basal, 8 (31%) were medial, 2 (8%) were apical, and 2 (8%) were medial–apical.

We examined the change in abnormal electrodes over time:

➢ Improvement was observed in 4 of the 22 OC failures (18.1%). Three of these showed complete normalization of impedance values, while one demonstrated partial reduction toward normal values without full normalization. All intraoperatively detected OC failures improved within the first month, while no improvement occurred in the remaining failures during the 12-month period.
➢ No improvement was detected during the follow-up period in any of the 4 SC failures (Table 2).

## 4. Discussion

The temporal pattern of impedance changes observed in this study is consistent with previous reports in the literature. Several studies have demonstrated that electrode impedance values typically increase following implantation, reach a peak at the time of first activation, and subsequently decline before stabilizing within the first postoperative months.

However, beyond confirming this well-established trend, the present study provides several additional clinically relevant contributions. First, the large cohort size and longitudinal design allow for a more precise characterization of impedance behavior over time in a predominantly homogeneous population using the CI422 electrode array. Second, this study offers a detailed analysis of the prevalence, anatomical distribution, and evolution of electrode malfunctions. Finally, the direct comparison between open-circuit and short-circuit failures provides novel insights into their differing clinical courses, particularly the limited recovery observed in SC cases compared to the partial reversibility of some OC cases.

Cochlear implantation is an effective treatment method that restores auditory input in patients with severe or profound hearing loss. It is also the first implantable cranial nerve implant [11]. With the increasing prevalence of implantation surgery, surgical complications are becoming more frequent. Electrophysiological measurements performed during surgery are of great importance in preventing these complications.

Despite numerous electrophysiological assessments being performed during and after cochlear implant surgery, the most commonly used test method is standard impedance telemetry. Tykocinski and colleagues have demonstrated that impedance telemetry provides detailed information not only about the implant array but also about changes in the intracochlear environment after implantation. However, although standard impedance telemetry provides important data about the status of cochlear implant electrodes and the intracochlear environment, it does not provide all the necessary information about neural responsiveness, spread of excitation, or functional activation of the auditory pathway, which require complementary measures such as ECAP/NRT or ESRT [12].

A review of the literature revealed numerous studies examining changes in impedance over time. In a study using Nucleus brand electrodes, a significant increase in electrode impedance was observed from the preoperative test to the first stimulation, followed by a decrease in impedance until the next follow-up. It has been reported that impedance values remained stable for approximately 2–4 months after the first stimulation [13]. While our findings regarding the temporal pattern of impedance changes are in agreement with previous studies, the stabilization observed around the 6-month time point in our cohort appears slightly later than the 2–4-month stabilization period reported in some earlier studies. This difference may be attributable to variations in follow-up protocols, electrode design, or cohort characteristics. In a similar study, Hughes et al. noted that electrode impedances changed during the first 3 months and stabilized by 6–8 months [14].

Consistent with our findings, the literature reports that postoperative measurements of electrode impedance values in children and adults show a significant increase compared to measurements taken during surgery. The electrode array is not stimulated between these two measurements, and the interval between implantation and the first stimulation is typically one month. Protein adsorption and tissue growth occur on the array during the first few weeks after implantation. Therefore, electrode impedance values are generally at their maximum during the first activation [15,16,17].

One of the most clinically relevant findings of this study is the differential behavior observed between open-circuit and short-circuit malfunctions. While a subset of OC cases demonstrated partial or complete improvement within the early postoperative period, no recovery was observed in SC cases during follow-up. This distinction may have important implications for clinical management, suggesting that OC abnormalities may in some cases represent transient or reversible conditions, whereas SC failures are more likely to indicate persistent structural or device-related issues.

In our study, impedances were monitored intraoperatively and at 1, 2, 6, and 12 months postoperatively in every individual who underwent implantation. We found that intraoperative impedances were significantly lower than postoperative values, consistent with previous reports. It was observed that impedances reached their maximum value at the 1-month follow-up, then showed a downward trend, and stabilized over the subsequent 6 months.

While these findings confirm previously reported impedance trends, the present study extends the existing literature in several important ways. Specifically, the large cohort size allows for more precise estimation of impedance behavior over time, and the combined analysis of longitudinal impedance changes together with the prevalence, anatomical distribution, and clinical course of individual electrode malfunctions may provide a more comprehensive characterization than has been available in prior studies. In particular, the differential evolution of open and short circuits observed in this cohort adds clinically relevant information regarding their expected natural history. Although the present study provides a detailed descriptive analysis of impedance trends and electrode malfunctions, further analytical approaches such as subgroup comparisons or predictive modeling were not feasible due to the limited number of malfunction events.

The significance levels of the average impedance values of electrode bundles grouped as basal, medial, and apical were investigated during the intraoperative and postoperative periods. This analysis allowed us to compare impedance trends across cochlear regions. Henkin et al.’s study found no significant difference between electrodes grouped as apical, medial, and basal [18]. In parallel with this result, our study also found that basal, medial, apical, and general impedance values showed the same rate of change intraoperatively and at 1, 2, 6, and 12 months postoperatively. The relatively smaller magnitude of impedance change observed in medial electrodes compared to basal and apical electrodes may reflect a more stable electrode–tissue interface in the middle cochlear turn. This region may be less affected by surgical trauma near the round window and less prone to apical fibrosis or osteoneogenesis, although this interpretation remains speculative and warrants further investigation. These regional differences should also be interpreted with caution, as they may be influenced by the specific design and insertion characteristics of the CI422 electrode array, and may not necessarily apply to other electrode configurations.

These regional differences and observed malfunction patterns may be partially explained by underlying biomechanical and biological factors. Basal electrodes may be more susceptible to abnormalities due to their proximity to the site of insertion at the round window or cochleostomy, where mechanical stress during electrode insertion is greatest. This region is also more prone to localized inflammatory responses, fibrosis, or early osteoneogenesis, which may contribute to alterations in the electrode–tissue interface and impedance characteristics.

In addition, the differing behavior of open-circuit and short-circuit failures may reflect distinct underlying mechanisms. Open-circuit abnormalities may in some cases be related to transient factors such as air bubbles, temporary fluid interface changes, or protein deposition on the electrode surface, which may resolve during the early postoperative period. In contrast, short-circuit failures are more likely to represent structural defects such as insulation breaches or direct electrical coupling between contacts, which are less likely to recover spontaneously. These interpretations are consistent with previous histopathological and engineering studies examining electrode–tissue interactions and device integrity. In contrast, short-circuit failures are more likely to represent structural defects such as insulation breaches or direct electrical coupling between contacts, which are less likely to recover spontaneously. These interpretations are consistent with previous histopathological and engineering studies examining electrode–tissue interactions and device integrity.

Impedance measurements are influenced by the electrode–tissue interface, the surrounding fluid environment, and the resistance of electrode contacts and lead wires; therefore, impedance abnormalities such as short circuit (SC) or open circuit (OC) may be encountered. In their study investigating the incidence of electrode failures, Shennawy et al. found that the total prevalence of devices with at least one abnormal electrode impedance value in intraoperative and postoperative measurements was 9% (4/44) [17]. Although our study did not assess functional hearing outcomes, prior reports suggest that electrode abnormalities may be associated with reduced auditory performance. Various studies have reported that OC is more common than SC in both intraoperative measurements and subsequent follow-ups. Carlson et al. found a higher incidence of OC (63.2%) compared to SC (37%) cases [19].

Electrode malfunctions can be caused by implant-related issues or manipulation during surgery. Faults can also occur during follow-up, but these are less common than those present during surgery [20]. One study showed that 66% of electrode faults occurred during surgery, 8% were present at initial activation, and the remainder occurred within the subsequent 12-month measurements [19].

In our study, at least one electrode malfunction was observed in 26 (7.2%) of the total 358 devices examined. Our observed malfunction rate of 7.2% is slightly lower than the rates reported in some previous studies, which have ranged between approximately 9% and 19.7%. This difference may be related to the relatively homogeneous electrode cohort in our study, predominantly consisting of CI422 devices, as well as potential variations in surgical technique, patient selection, and definitions of electrode malfunction across studies. Of the 26 devices with electrode malfunctions, 22 (84.7%) had open circuits (OCs) and 4 (15.3%) had short circuits (SCs). In our study, improvement was observed in only 4 of the OC electrodes during the first postoperative month. This improvement may be explained by the elimination of temporary factors that may cause OC malfunction during surgery. The 4 SC electrodes recorded intraoperatively were found to remain unchanged until the 12th postoperative month. It should be noted that the overall prevalence of electrode malfunctions observed in this study (7.2%) reflects the performance characteristics of a predominantly CI422 electrode cohort and should therefore be interpreted as device-specific rather than universally generalizable across different implant systems. As in our study, a detailed analysis of fault types is important for predicting recovery rates over time. These abnormalities have been reported to be associated with changes in hearing performance in prior studies. In the present study, although functional auditory outcomes were not evaluated, impedance telemetry proved useful for documenting electrode integrity intraoperatively and for monitoring longitudinal changes during follow-up and programming sessions, thereby helping to identify persistent or evolving abnormalities [19,21,22].

Previously published reports have found that multiple circuit failures may indicate deterioration in device performance that could necessitate explantation. A decline in sensory performance has been detected, and it has been suggested that multiple short circuits or open circuits within a series may predict critical impairments in future implant function. Based on their experience, the authors consider reimplantation if three or more electrodes fail [23,24,25]. However, since the number of electrodes can vary depending on the brand, precise numerical data should not be used. In our study, a small number of patients presented with multiple electrode malfunctions; however, revision surgery was not indicated because their auditory performance remained satisfactory.

From a clinical perspective, the findings of this study provide several practical implications for patient management and device programming. First, the observed stabilization of impedance levels after approximately 6 months suggests that major programming adjustments could be considered at or after this time point, when impedance fluctuations have decreased.

Second, in cases where an open-circuit abnormality is detected intraoperatively, clinicians may consider informing patients and their families about the possibility of spontaneous improvement, as approximately 18% of OC cases in this cohort showed partial or complete recovery within the first postoperative month. In contrast, the absence of recovery in short-circuit failures suggests that these abnormalities are likely to persist, and early deactivation of the affected electrodes during initial programming may be considered to optimize device performance and avoid unnecessary stimulation artifacts.

A major limitation of this study is the extreme homogeneity of the electrode types included. Specifically, 94% of the implanted devices consisted of a single lateral wall electrode array (Cochlear CI422, Slim Straight). Therefore, the findings of this study are primarily applicable to this specific electrode model. The impedance behavior and electrode malfunction patterns reported here may not generalize to newer generation electrode designs, such as perimodiolar, slim modiolar, or mid-scala arrays, nor to devices from other manufacturers. Consequently, the generalizability of these findings to broader cochlear implant populations is limited, and the results should be interpreted as device-specific observations.

This study has several limitations inherent to its retrospective design. In addition, standard deviation values were not available in the summarized dataset used for this analysis; therefore, impedance values are presented as mean values only. Although demographic and device-related variables such as age, implant side, and electrode model were available and reported descriptively, they were not included in a multivariate or predictive analysis due to the relatively small number of electrode malfunctions. In addition, speech perception outcomes and functional hearing measures were not analyzed, limiting the ability to assess the clinical impact of impedance abnormalities. In addition, due to the relatively low number of electrode malfunctions observed in this cohort (*n* = 26), further subgroup analyses or multivariate modeling to identify independent predictors of electrode failure were not performed. Such analyses would likely be underpowered and potentially yield unreliable results. Future studies with larger numbers of malfunction cases are needed to better identify potential risk factors and predictors of electrode abnormalities. The study population included a wide age range, with a predominantly pediatric cohort. Although age-related differences in impedance behavior and electrode malfunction patterns may be clinically relevant, such analyses were not performed in the present study. This was primarily due to the limited number of malfunction events and the imbalance in age distribution, which would reduce the reliability of subgroup comparisons. Future studies specifically designed to investigate age-related effects may provide further insight into these potential differences.

Furthermore, this single-center study, which examined 358 implants, has a larger sample size than most reports in the literature. These findings may add complementary data to the existing literature in terms of both sample size and results.

## 5. Conclusions

This study suggests that impedance values tend to peak at the first postoperative month and subsequently stabilize after approximately 6 months, indicating a potential time window for clinical monitoring and device optimization. These findings support the importance of regular follow-up measurements for individualized programming and ongoing clinical assessment.

While similar temporal trends have been reported in previous studies, the present study adds to the existing literature by providing a large-scale longitudinal characterization of impedance behavior, including regional variations across cochlear segments and detailed observations on the prevalence and course of electrode circuit abnormalities. These findings may contribute to a more comprehensive understanding of impedance dynamics, particularly during the early postoperative period.

However, the results should be interpreted in the context of the predominantly CI422 electrode cohort and may not be directly generalizable to other implant designs. Further studies incorporating functional auditory outcomes and larger, more diverse populations are needed to better understand the clinical implications and potential predictors of electrode malfunctions.

## Figures and Tables

**Table 1:** Impedance changes (kΩ) according to electrode positions.

	Intraoperative	1st Month	2nd Month	6th Month	12th Month	*p* Value
**Basal**	12.1	15.3	13.1	10.8	10.6	<0.05
**Medial**	11.2	13.4	12.2	11.6	11.4	<0.05
**Apical**	14.2	17.0	15.6	11.2	11.0	0.459
**Total**	12.5	15.4	13.3	13.1	11.1	0.007

**Table 2:** Change in Abnormal Electrodes Over Time.

Patient	Fault Type	Defective Electrode Number	Defective Electrode Position	Defective Electrode Condition
1	OC	7	Basal	improvement
2	OC	14	Medial	no change
3	OC	1,2,3	Basal	no change
4	OC	16,17	Apical	no change
5	OC	10,16,18,20,21,22	Medial, apical	improvement (10)
6	OC	20	Apical	no change
7	OC	4	Basal	no change
8	OC	11	Medial	no change
9	OC	4	Basal	no change
10	OC	1	Basal	improvement
11	OC	8	Medial	no change
12	OC	1,2,3	Basal	no change
13	OC	9	Medial	no change
14	SC	4,5	Basal	no change
15	OC	1	Basal	improvement
16	OC	6	Basal	no change
17	OC	7,4	Basal	no change
18	SC	6,7	Basal	no change
19	OC	9	Medial	no change
20	OC	4	Basal	no change
21	OC	2	Basal	no change
22	SC	11,12	Medial	no change
23	OC	3,5	Basal	no change
24	OC	8,13	Medial	no change
25	OC	12,14,16,20	Medial, apical	no change
26	SC	10,11,12,13,14	Medial	no change

## Data Availability

No new data were created or analyzed in this study.
